# The impact of vitamin D on type 2 diabetes management: boosting PTP1B gene expression and physical activity benefits in rats

**DOI:** 10.1186/s12263-023-00736-z

**Published:** 2024-03-02

**Authors:** Kimya Khaledi, Rastegar Hoseini, Ahmad Gharzi

**Affiliations:** 1 Department of Exercise Physiology, Faculty of Sport Sciences, Razi University, Kermanshah, Iran; 2 Department of Biology, Faculty of Science, Razi University, Kermanshah, Iran

**Keywords:** PTP1B, Irisin, Vitamin D, Aerobic training, Type 2 diabetes

## Abstract

**Background:**

The protein tyrosine phosphatase 1B (PTP1B) plays a crucial role in the development of insulin resistance. Aerobic training (AT) and vitamin D (Vit D) supplementation have been shown to individually improve glucose tolerance and diabetes-related factors. However, the impact of their combined effect on PTP1B gene expression and serum irisin in the visceral adipose tissue remains unknown. This study aims to investigate whether 8 weeks of combined AT with Vit D supplementation can improve the expression of PTP1B in adipose tissue and serum irisin in obese rats with type 2 diabetes (T2D).

**Methods:**

Fifty male Wistar rats were divided into two groups: diabetic (*n* = 40) and non-diabetic (ND; *n* = 10). The diabetic rats were further divided into four groups: aerobic training with vitamin D supplementation (D + AT + Vit D; *n* = 10), aerobic training only (D + AT; *n* = 10), vitamin D supplementation only (D + Vit D; *n* = 10), and control (D + C; *n* = 10). The D + Vit D and D + AT + Vit D groups received 5000 IU of vitamin D via injection once a week, while the D + AT and D + C groups received sesame oil. Diabetes was induced in all groups except the nondiabetic group by intraperitoneal (IP) injection of streptozotocin. At the end of the intervention, blood and adipose tissue samples were collected, and RNA was extracted from adipose tissue for real-time PCR analysis of PPTP1B gene expression.

**Results:**

There was an increase in serum Vit D and irisin levels and a decrease in HOMA-IR and PTP1B gene expression in the diabetic rat model treated with D + AT and injected with 50,000 IU/kg/week of Vit D. Comparatively, when treated with D + AT + Vit D, the downregulation of PTP1B was significantly higher (*p* = 0.049; *p* = 0.004), and there was a significant increase in irisin (*p* = 0.010; *p* = 0.001).

**Conclusion:**

The present study shows that the combined AT and Vit D supplementation positively impacts the expression of PTP1B in adipose tissue and serum irisin in rats with T2D. These findings suggest that combining AT with Vit D supplementation can provide a new and effective strategy to improve glucose tolerance and diabetes-related factors in individuals with T2D by regulating the expression of PTP1B in adipose tissue and promoting the synthesis of beneficial irisin protein.

## Introduction

Type 2 diabetes (T2D) is a metabolic condition characterized by hyperglycemia resulting from a combination of insufficient insulin secretion and resistance to insulin action [1]. The Iranian adult population has a prevalence of almost 8% of T2D [2], and approximately 258 million cases were diagnosed with T2D in 2010 worldwide [3]. Genetic mutations and heredity, as well as obesity and obesity-related abnormalities, are among the factors that contribute to the pathogenesis of T2D [4]. Although the disease has multifactorial origins, obesity is one of the primary risk factors that can lead to insulin resistance and the development of T2D. This correlation has been supported by previous research studies [4, 5].

Current research emphasizes identifying genetic factors that contribute to obesity and T2D. Recently, several genetic factors that contribute to the prevalence and severity of both type 1 and type 2 diabetes have been recognized. Impaired expression or polymorphisms of these genes can result in damaged insulin secretion and function [6]. Protein tyrosine phosphatase 1B (PTP1B), a key member of the PTP family, has been identified as a negative regulator of insulin signaling [7] and a potential therapeutic target for T2D [8]. PTP1B has been shown to cause interference with insulin receptor substrate-1 (IRS-1), thereby impairing glucose uptake by degradation of insulin signaling pathways [7]. On the other hand, elevated serum levels of irisin, an important regulator of insulin signaling, have been associated with increased ER (endoplasmic reticulum) stress leading to insulin resistance. This connection has been established through several studies [9, 10]. Irisin is a myokine that is triggered by exercise and is believed to increase energy expenditure by brown adipose tissue [11]. Studies suggest that the browning process could be in response to physical activity and a cold environment, with the upregulation of peroxisome proliferator-activated receptor-gamma (PPARγ) coactivator 1 alpha (PGC-1α) leading to increased expression of the fibronectin type 3 domain containing 5 (FNDC5) gene, which encodes the precursor protein for irisin production [12, 13]. Studies suggest that inhibition of PTP1B and increased serum levels of irisin can lead to improvement in insulin resistance, as well as insulin and blood glucose levels [8, 10]. Several studies have investigated the effects of aerobic training (AT), either alone or in combination with diet management, on hormonal and genetic factors that impact insulin function in nondiabetic and diabetic subjects [14, 15]. Additionally, while some studies have shown that AT can have a significant effect on the expression or protein levels of genes that impact insulin secretion and function, such as transcription factor 7-like 2 (TCF7L2) [4], forkhead box O1 (FOXO1) [16], and PPARγ [17], the direct effect of AT on PTP1B expression in adipose tissue and serum irisin levels in diabetic patients remains understudied. Along with various suggested strategies for metabolic control of diabetes [14, 15], the use of antioxidants like vitamins E, A, and C and coenzyme Q10 have also been proposed [18]. The use of vitamin D (Vit D) to decrease metabolic profiles [19], inflammatory factors [20], and biomarkers of oxidative stress has recently gained attention [21, 22]. Vit D appears to improve insulin sensitivity, which in turn influences metabolic profiles [22]. Limited research is available on the effects of Vit D on the metabolic profiles of diabetic patients. In rats, a diet supplemented with Vit D has been shown to significantly delay the progression of glucose intolerance, hyperglycemia, hyperinsulinemia, dyslipidemia, and oxidative stress [17]. Previous studies have mainly investigated the individual effects of AT and Vit D on glucose tolerance and diabetes-related factors in skeletal muscle. To our knowledge, limited reports exist regarding the effects of combined AT and Vit D on PTP1B expression in adipose tissue and serum irisin levels. While previous research has established the benefits of separate AT and Vit D supplementation, the long-term effects of their combination on PTP1B gene expression in visceral adipose tissue and serum irisin remain unclear. Therefore, the purpose of this study was to investigate the effect of an 8-week combined AT and Vit D supplementation on PTP1B gene expression and serum irisin levels in the visceral adipose tissue of obese rats with T2D.

## Methods

### Ethical approval

This study followed the scientific protocol, being randomized, single blinded, and placebo controlled. Registration details of the study can be found on the Iranian Registry of clinical trials at http://www.irct.ir with the identifier number IRCT20220510054809N1. The study design and conduction were under the approval and supervision of the Research Ethics Committee of Razi University (no. IR.RAZI.REC.1401.013), Iran. The care, maintenance, and sacrifice of animals were conducted in adherence with the Danish “Animal Welfare Act” (LBK 1343 of 04/12/2007).

### Animals

In this experimental research, a total of 50 Wistar rats were used. These rats were adult males, aged 4–5 weeks, and weighed approximately 180 ± 10 g. They were procured from laboratory animals, Kermanshah, Iran. The rats were categorized into two main groups, comprising 40 diabetic rats and 10 nondiabetic (ND) rats. The diabetic rats were induced with diabetes using intraperitoneal streptozotocin (STZ) and nicotinamide along with a high-fat diet. The 40 diabetic rats were further split into four subgroups: AT with Vit D supplementation (D + AT + Vit D; *n* = 10), AT only (D + AT; *n* = 10), Vit D supplementation only (D + Vit D; *n* = 10), and control (D + C; *n* = 10). The rats in the D + Vit D and D + AT + Vit D groups were given 5000 IU of Vit D by injection once a week, while rats in the D + AT and D + C groups were given sesame oil instead. Additionally, the D + AT and D + AT + Vit D groups underwent an 8-week AT routine, 5 days a week. All rats were kept in transparent polycarbonate cages. The cages were 30 cm in length and 15 cm in width and height. The animals were maintained under a 12-h light/dark cycle, with a temperature of 25 ± 2 °C and humidity of 45–55% [23, 24]. They had free access to food and water in 500-ml bottles, and ethical principles of working with laboratory animals were followed throughout the study.

### Obesity induction and diabetes induction method

The male Wister rats used in the study were acclimatized to a new environment for 2 weeks before beginning the intervention program. These rats were given a high-fat diet in the form of pellets, which contained standard mouse food powder (365 mg/kg), sheep fat (310 mg/kg), mixed vitamins and minerals (60 mg/kg), DL methionine (3 mg/kg), yeast powder (1 mg/kg), and chloride sodium (1 mg/kg) to increase their weight. Once their weight exceeded 300 g after 2 weeks, the rats were induced with diabetes by injecting 60 mg/kg BW of streptozotocin dissolved in 0.1-M citrate buffer with pH = 4.5, followed by 110 mg/kg BW of nicotinamide after 15 min intraperitoneally. The induction of T2D was confirmed by measuring blood sugar using a glucometer 2 weeks later. Blood samples were collected from the lateral tail vein after immersing it in 42 °C water for 40–50 s, and blood sugar above 200 mg/l was considered an indicator of diabetes [25]. No insulin treatment was provided to the animals throughout the study.

### Intervention programs

#### Vitamin D supplementation

The study included two groups, D + AT + Vit D and D + Vit D, which received 5000 international units (IU) of Vit D once a week through a single-dose injection. In contrast, the D + AT and D + C groups were injected with sesame oil instead of Vit D. To measure Vit D serum concentration, a Vit D enzyme-linked immunosorbent assay (ELISA) rat kit from Immune Diagnostics System Ltd. in Boldon, UK, was used, which had an intraassay coefficient of variation of 1.63% and method sensitivity of 1.33 mg/dL [17].

### Exercise protocol

**Fig. 1:**
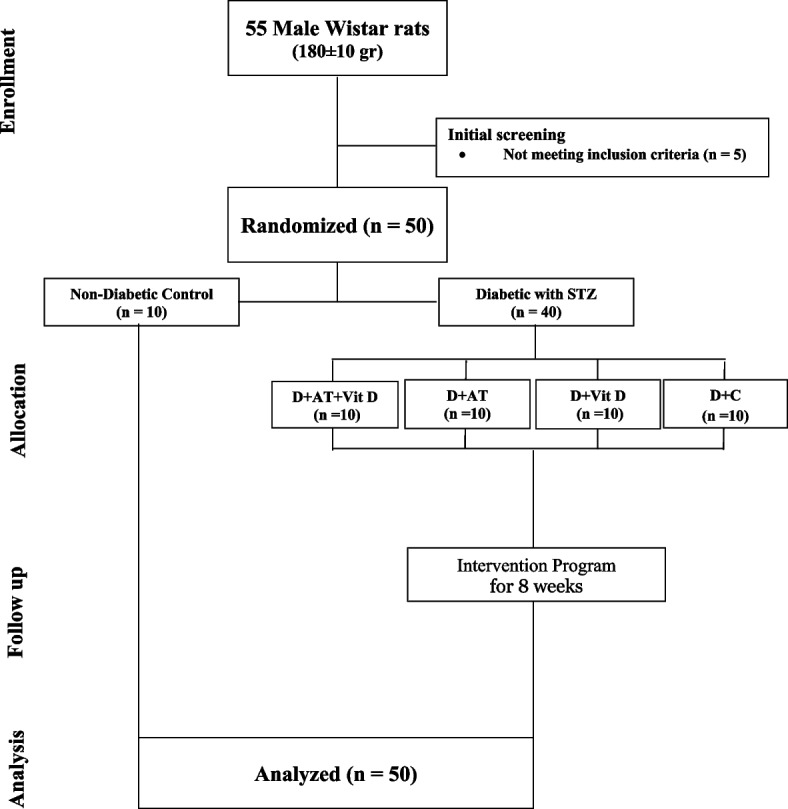
Diagram of the progress through the phases of the parallel randomized trial of four groups (enrolment, intervention allocation, follow-up, and data analysis). D + AT + Vit D, diabetic + aerobic training + vitamin D supplement; D + AT, diabetic + aerobic training; D + Vit D, diabetic + vitamin D supplement; D + C, diabetic + control; NC, nondiabetic control

Following diabetes induction, the rats were separated into 4 groups of 10 each, randomized based on body weight. A week before the main AT sessions, rats were subjected to a familiarization AT session on a zero-incline treadmill. The session involved running for 5 min at a speed of 8–10 m/min 5 days a week. The actual AT program spanned over 8 weeks on a zero-incline treadmill with a speed range of 15–25 m/min, for 30–60 min each day, 5 days a week. In the first week, rats ran on the treadmill for 30 min at a speed of 15 m/min, with the AT intensity and duration gradually increasing to reach 25 m/min and 60 min, respectively, by the end of the 8th week. The training intensity equaled 50–60% of VO_2max_ [26]. For both pre- and post-AT, rats were warmed up for 10 min at 5 m/min and cooled down for 10 min. Rats that had stopped running were stimulated to continue running via a combination of sound (from striking the rotating tape wall) and low-voltage electric stimuli. The use of the latter was discontinued after the first week in compliance with laboratory animal ethical guidelines [23, 24] (Fig. 1).

### Measurement variables

#### Food intake, body weight, and body mass index

At weekly intervals between 9:00 and 11:30 a.m., all animals were weighed using a scale. The nose-to-anus length was measured to calculate the body mass index (BMI). To measure food intake (FI), the weight of uneaten food was subtracted from the total amount (20 g/day) of food given in each cage.

#### Blood sampling and RNA extraction/real-time PCR

To eliminate the immediate effects of training and to control for uncontrollable variables caused by the training program, animals were anesthetized 48 h after the last training session (46, 15) through an intraperitoneal injection of ketamine (70 mg/kg BW) and xylazine (3–5 mg/kg BW) while considering ethical principles. Blood samples were taken from the vena cava, and plasma concentrations of glucose and insulin were measured via the Hitachi Auto Analyzer (type 7170; Hitachi Electronics, Hitachi, Japan). The homeostatic model assessment for insulin resistance (HOMA-IR) score was calculated by using glucose and insulin concentrations, as follows:

$$\mathrm{HOMA}-\mathrm{IR}=\,\left(\mathrm{glucose\,concentration\,}\times\,\mathrm{\,insulin\,concentration}\right)\div\,22.5$$

The concentration of irisin was quantified through the use of an ELISA kit manufactured in China, possessing a sensitivity of 0.15 mg/l. Additionally, an ELISA kit was employed to determine the serum concentration of serum 25-hydroxyvitamin D. Furthermore, the visceral adipose tissue was rapidly separated and weighed, washed in saline to prevent RNA degradation, transferred to tubes containing RNA Later, placed in liquid nitrogen, and then preserved in a refrigerator maintained at − 80 °C for subsequent assessments. The RNA present in 20 mg of adipose tissue was isolated using a combined method involving TRI Reagent® (MRC Inc., USA) and miRNeasy techniques (Viragene, Iran). Initially, the frozen tissue was homogenized with a gentleMACS™ Octo Dissociator system and M tubes (Viragene, Iran) using the RNA_02 program along with 2 ml of TRI Reagent® buffer, following the manufacturer’s guidelines. The samples were then allowed to incubate at room temperature for 5 min, followed by centrifugation at 12,000 g at 4 °C for 10 min. After, the desired layer was pipetted into a 1.5-ml tube and mixed with 400 μl of chloroform, followed by a further centrifugation step at 12,000 g at 4 °C for 30 min and keeping the sample at room temperature for 3 min. Next, the upper layer was thoroughly mixed with ethanol by inverting the tube and loaded onto a miRNeasy spin column (Viragene, Iran) according to the manufacturer’s protocol. Finally, RNA was eluted in 30 μl of RNAse-free water. A PrimeScript RT reagent kit (Viragene, Iran) was used for reverse transcription into cDNA using 1 μg of total RNA. TB Green Premix Ex-Taq II (TaKaRa, Dalian, China) was used for quantitative RT-PCR. The expression levels of target genes were calculated using the comparative 2 − ΔΔct method. The Viragene kit from Iran was used to measure the expression of the PTP1B gene using the protein tyrosine phosphatase non-receptor type 1 (NM_002827.4) as a reference gene, and it possessed a sensitivity of 0.034. The sequence of primers utilized for PTP1B can be found in Table 1. All animal testing methods were conducted following the fundamental principles of the 2008 Helsinki Declaration.

**Table 1:** The forward and reverse  primer sequences

Genes	Primers	
**PTP1B**	Forward	GCAGTTGGAGTTGGAGAACCTG
	Reverse	CGTGCTCTGGGCTGAGTG

### Statistical analysis

The normal distribution of the data was examined using the Shapiro–Wilk test. The data was then analyzed utilizing the SPSS 26.0 program (SPSS, Inc., Chicago, IL, USA) through paired sample *t*-test and one-way ANOVA along with a Tukey post hoc test. Values of *p* < 0.05 were considered statistically significant.

## Results

Table 2 presents the average body weight, FI, and BMI. Following an 8-week intervention, there was a noticeable difference between groups for the average BW, FI, and BMI. The D + AT + Vit D, D + AT, and D + Vit D groups indicated statistically significant reductions in BW, FI, and BMI when compared to the baseline values (*p* < 0.01). In contrast, the D + C and ND groups reported significant increases in the mean BW, FI, and BMI towards the end of the study in comparison to the commencement of the study.

**Table 2:** Comparison of mean ± SD of body weight, FI, and BMI before and after intervention

**Variables**	**D + AT + Vit D**	**D + AT**	**D + Vit D**	**D + C**	**NC**	***p*-value**^a^
**Body weight (g)**						
**Before**	305.70 ± 2.90	308.10 ± 2.80	303.80 ± 2.78	304.70 ± 1.88	207.60 ± 3.80	0.001^**¥**^
**After**	250.50 ± 5.79	263.80 ± 2.78	286 ± 3.19	322.90 ± 3.60	213.50 ± 4.35	
**Δ**	− 55.20 ± 2.89**α¥€£**	− 44.30 ± 0.02**¥€£**	− 17.80 ± 0.41**€£**	− 18.20 ± 1.72**£**	5.90 ± 0.55	
**P†**	0.001*	0.001*	0.001*	0.001*	0.009*	
**FI (g/day)**						
**Before**	15.17 ± 0.026	15.30 ± 0.029	15.14 ± 0.018	15.18 ± 0.024	14.64 ± 0.048	
**After**	15.11 ± 0.02**4**	15.23 ± 0.033	15.11 ± 0.025	15.61 ± 0.045	14.70 ± 0.030	0.002**¥**
**Δ**	− 0.06 ± 0.02**€£**	− 0.07 ± 0.04**€£**	− 0.03 ± 0.07**€£**	0.7 ± 0.01**£**	0.06 ± 0.018	
**P†**	0.002*	0.002*	0.002*	0.001*	0.023*	
**BMI (kg/m**^**2**^**)**						
**Before**	0.74 ± 0.033	0.76 ± 0.019	0.77 ± 0.021	0.76 ± 0.030	0.52 ± 0.016	0.011**¥**
**After**	0.61 ± 0.015	0.65 ± 0.011	0.71 ± 0.026	0.80 ± 0.016	0.53 ± 0.014	
**Δ**	− 0.13 ± 0.018**α¥€£**	− 0.11 ± 0.008^**¥€£**^	− 0.06 ± 0.005**€£**	0.04 ± 0.014	0.01 ± 0.002	
**P†**	0.001*	0.001*	0.005*	0.006*	0.013*	

*D* + *AT* + *Vit D* Diabetic + aerobic training + vitamin D supplement, *D* + *AT* Diabetic + aerobic training, *D* + *Vit D* Diabetic + vitamin D supplement, *D* + *C* Diabetic + control, *NC* Nondiabetic control

^*^Data analysis was done by the analysis of one-way analysis of variance test followed by post hoc Tukey’s test. P†Statistical analysis was done by paired sample *t*-test. *Significantly different in comparison pre- and post within the groups. ¥Significantly different comparing Δ between groups

αSignificantly different compared to AT. ¥Significantly different compared to vit D. €Significantly different compared to C. £Significantly different compared to NC

**Fig. 2:**
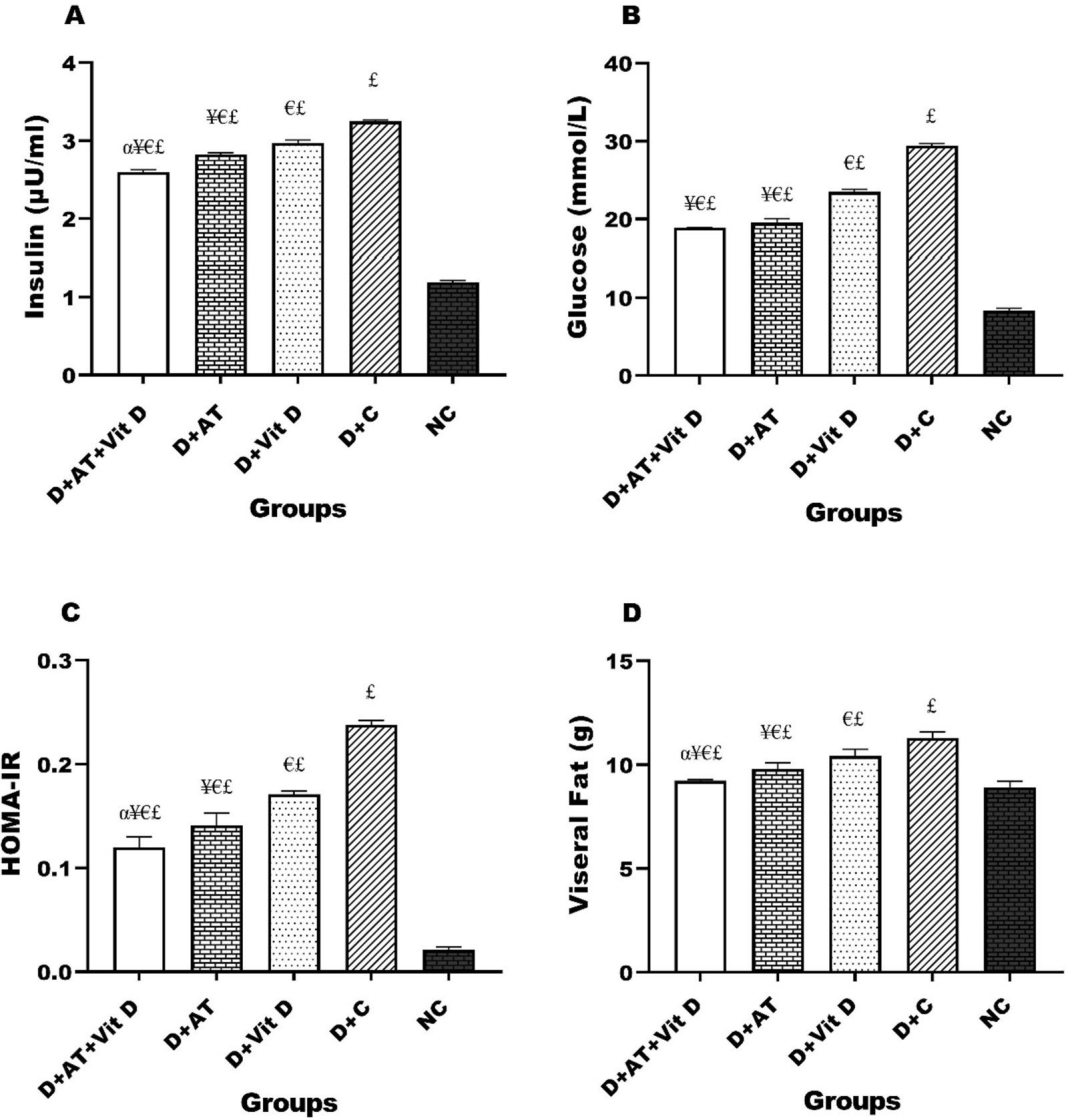
Comparison of mean ± SD of insulin, glucose, HOMA-IR, and visceral fat after the intervention among the groups. D + AT + Vit D, diabetic + aerobic training + vitamin D supplement; D + AT, diabetic + aerobic training; D + Vit D, diabetic + vitamin D supplement; D + C, diabetic + control; NC, nondiabetic control. αSignificantly different compared to AT. ¥Significantly different compared to Vit D. €Significantly different compared to C. £Significantly different compared to NC

Figure 2 (A, B, C, and D) presented statistically significant differences in insulin, glucose, HOMA-IR, and visceral fat among the groups, with the lowest levels observed in the ND group and the highest in the D + C group. Additionally, D + AT + Vit D, D + AT, and D + Vit D resulted in lower levels of insulin, glucose, HOMA-IR, and visceral fat compared to the D + C group. The results showed that D + AT + Vit D showed significantly lower insulin (*p* = 0.003), HOMA-IR (*p* = 0.012), and visceral fat (*p* = 0.001) when compared to D + AT. There were no significant differences in blood glucose levels between D + AT + Vit D and D + AT (*p* = 0.091); however, a significant difference was noted in insulin (*p* = 0.014), glucose (*p* = 0.008), HOMA-IR (*p* = 0.001), and visceral fat (*p* = 0.001) when comparing the D + AT + Vit D group to D + Vit D (*p* < 0.05 for all three variables). Moreover, D + AT induced a more significant decrease in insulin (*p* = 0.026), glucose (*p* = 0.006), HOMA-IR (*p* = 0.001), and visceral fat (*p* = 0.007) than D + Vit D. The ND group showed significant differences in insulin, glucose, HOMA-IR, and visceral fat when compared to the other groups (*p* < 0.05 for all three variables).

**Fig. 3:**
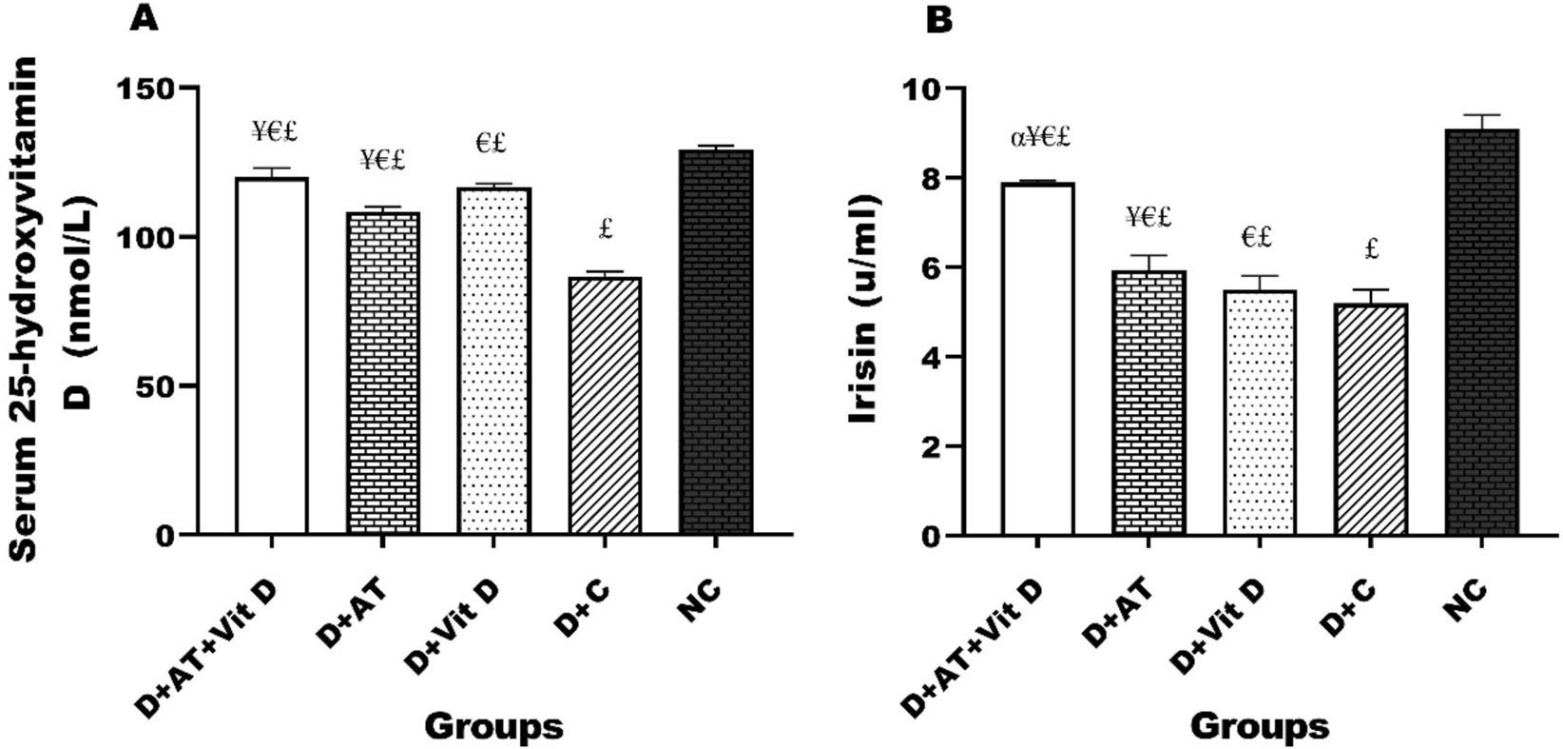
Comparison of mean ± SD of serum 25-hydroxyvitamin D and irisin after the intervention among the groups. D + AT + Vit D, diabetic + aerobic training + vitamin D supplement; *D* + *AT*, diabetic + aerobic training; *D* + *Vit D*, diabetic + vitamin D supplement; *D* + *C*, diabetic + control; *NC*, nondiabetic control. Values were calculated using a one-way analysis of variance followed by post hoc Tukey’s test. αSignificantly different compared to AT. ¥Significantly different compared to Vit D. €Significantly different compared to C. £Significantly different compared to NC

The results in Fig. 3 indicate a statistically significant difference in serum 25-hydroxyvitamin D and serum irisin levels among the groups, with the highest levels observed in the ND and D + AT + Vit D groups and the lowest level in the D + C group. D + AT + Vit D, D + AT, and D + Vit D led to increased levels of serum 25-hydroxyvitamin D and serum irisin compared to the D + C group. The results showed that D + AT + Vit D significantly increased serum 25-hydroxyvitamin D (*p* = 0.006; *p* = 0.001) compared to D + AT and D + C, respectively. No significant difference was observed in the serum 25-hydroxyvitamin D levels between D + AT + Vit D and D + Vit D (*p* = 0.286). Additionally, D + AT + Vit D significantly upregulated serum irisin (*p* = 0.002; *p* = 0.001, *p* = 0.001 compared to D + AT, D + Vit D, and C, respectively. Furthermore, D + Vit D induced a more significant increase in the serum 25-hydroxyvitamin D (*p* = 0.001) than D + AT, while D + AT significantly upregulated serum irisin (*p* = 0.011) compared to D + Vit D. There was a significant difference in the levels of serum 25-hydroxyvitamin D and serum irisin observed between the ND group and the other groups (*p* < 0.05 for all three variables).

**Fig. 4:**
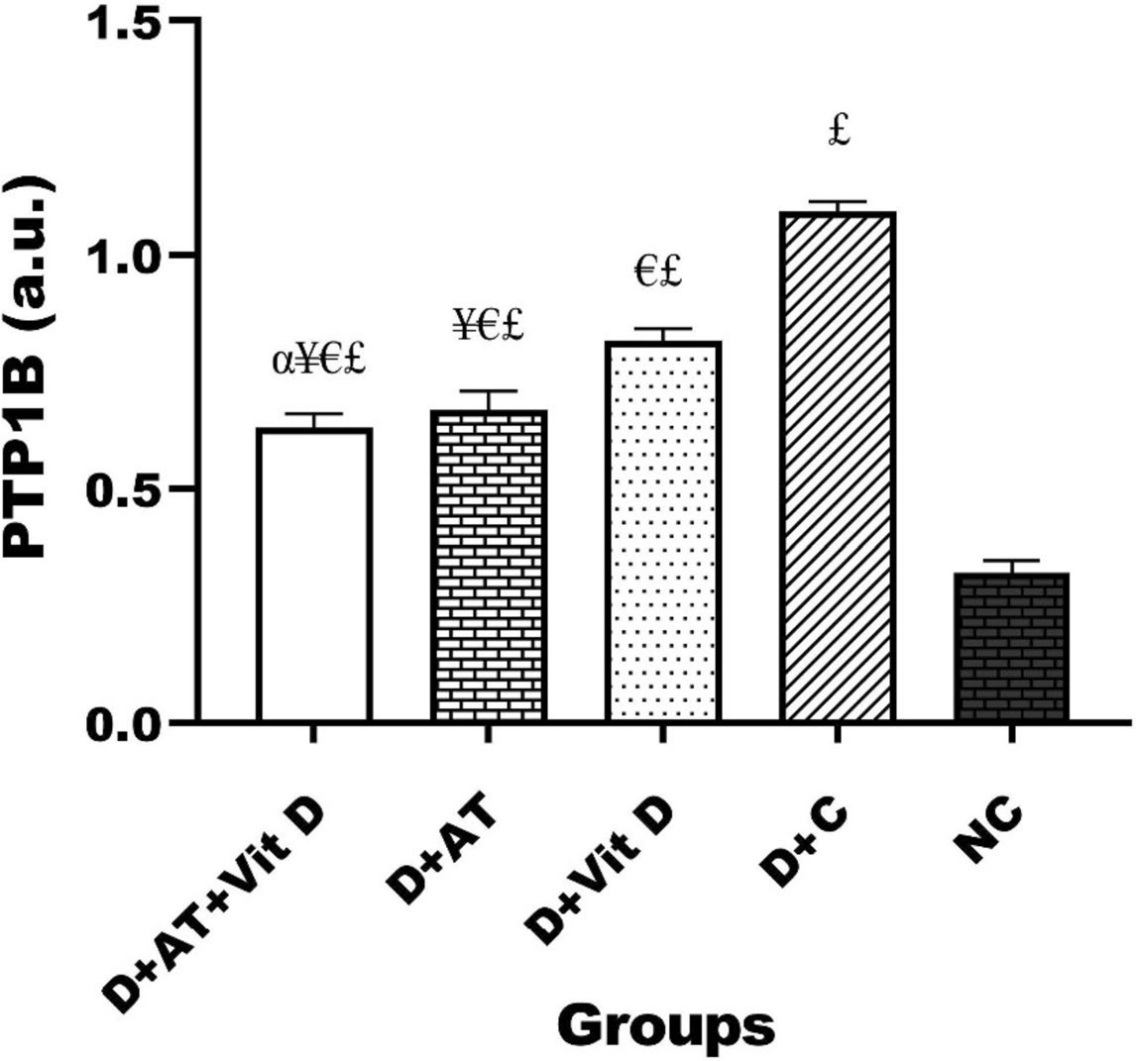
Comparison between mean ± SD of PTP1B gene expression between groups. D + AT + Vit D, diabetic + aerobic training + vitamin D supplement; D + AT, diabetic + aerobic training; D + Vit D, diabetic + vitamin D supplement; D + C, diabetic + control; NC, nondiabetic control. Value was calculated using a one-way analysis of variance followed by post hoc Tukey’s test. αSignificantly different compared to AT. ¥Significantly different compared to Vit D. €Significantly different compared to C. £Significantly different compared to NC

Figure 4 displays the results of PTP1B gene expression. A significant difference in PTP1B gene expression was observed among all groups, as determined by one-way ANOVA. Furthermore, D + AT + Vit D, D + AT, and D + Vit D resulted in decreased levels of PTP1B compared to D + C. Based on the results, D + AT + Vit D significantly downregulated PTP1B when compared to D + AT and D + Vit D (*p* = 0.049; *p* = 0.004), respectively. Also, D + AT induced a more significant decrease in PTP1B (*p* = 0.001) compared to D + Vit D. Additionally, there was a significant difference in PTP1B gene expression observed between the ND group and the other groups (*p* < 0.05 for all three variables).

## Discussion

Our study demonstrated that in rats with T2D, combining AT with Vit D for 8 weeks resulted in significant improvements in measures including body weight (BW), body mass index (BMI), visceral fat, insulin, fasting blood glucose, and HOMA-IR. In comparison, separate AT and Vit D treatments showed less improvement. The D + C group showed a significant increase in several measures including BW, BMI, visceral fat, insulin, fasting blood glucose, and HOMA-IR when compared to other groups. Diabetic patients often exhibit insulin resistance, dyslipidemia, and increased systemic inflammation and oxidative stress, which can result in various complications such as diabetic retinopathy, cardiovascular disease (CVD), hypertension, neuropathy, and nephropathy [27, 28]. Previous studies have examined the impact of AT and Vit D on insulin, fasting blood glucose, and HOMA-IR in human subjects with T2D [29] and elderly women with nonalcoholic fatty liver disease (NAFLD) [30]. Similar investigations have also been conducted in animal models, and the outcomes of this study were consistent with other studies [17, 31]. For instance, a study by Hoseini et al. demonstrated that administering AT and Vit D to obese rats improved BW, BMI, and insulin resistance after 8 weeks [32]. Similar results have been reported in multiple experimental studies [33, 34]. The mechanisms by which AT may result in these improvements include increased insulin receptor signaling, increased glucose transport protein-4 (GLUT-4), increased muscular capillaries and mitochondria, and glucose uptake, resulting in increased glycogen synthase and hexokinase enzyme activity [35]. AT also improves free fatty acid homeostasis [36] in serum and muscle tissue, uses it as a metabolic substrate, and reduces body weight and visceral fat [37]. Vit D has been shown to boost insulin production in the pancreas by triggering Vit D receptors (VDR) that are present in the pancreas and skeletal muscle cells [38]. It also enhances the amount of GLUT 4 by working on the skeletal muscle cells [39]. Additionally, Vit D reduces the concentration of inflammatory factors that lead to insulin resistance by suppressing the nuclear factor kappa-light-chain-enhancer of activated b cells (NF-Κb) gene expression [40, 41]. The study found significantly higher levels of serum irisin in the experimental groups compared to the control group after 8 weeks, with the AT + Vit D group experiencing a significantly higher increase than the AT and Vit D alone. The overexpression of irisin in obese mice has been linked to improved insulin sensitivity, enhanced energy expenditure, and reduced hyperlipidemia, hyperglycemia, and hypertension [42]. Irisin treatment of muscle cells stimulates glucose and fatty acid uptake, resembling the metabolic impact of insulin. It also increases the expression of genes such as GLUT4 and PPARalpha, which are involved in glycogenolysis and gluconeogenesis, respectively. In obese individuals, the production of irisin and FNDC5 is increased, enhancing glucose absorption by muscle cells and preventing high blood sugar levels [10, 43]. However, in individuals with diabetes, the amount of FNDC5 in muscle cells decreases by approximately 15%. Studies have confirmed that individuals with prediabetes or T2D have lower levels of irisin [44, 45]. The inflammatory response has been identified as a possible contributing factor to decreased levels of irisin. The precise biological mechanism underlying the higher levels of irisin in obesity and reduced irisin secretion in diabetes remains unclear.

Several previous studies have shown that both adipose and muscle tissues contribute significantly to the elevation of serum irisin levels after exercise training [46, 47]. Irisin levels increase briefly following an exercise session and can reduce both the weight of obese individuals and the insulin resistance of diabetic patients [48]. High levels of serum irisin have been associated with a lower risk of high BMI and coronary atherosclerosis in different population studies [49, 50]. Short-term exercise-induced irisin elevation leads to various positive metabolic changes [51, 52]. Lipid tissue is responsible for the baseline level of irisin in the blood of obese individuals [53]. Recent studies have highlighted the presence of the Vit D receptor in β cells of the pancreatic tissue, with variations in genes associated with Vit D metabolism and VDR expression linked to the pathogenesis of T1DM and T2D [53, 54]. Vit D-deficient mice have impaired insulin secretion to glucose stimulation, which can be improved upon administration of Vit D3. Moreover, Vit D has been observed to have a beneficial effect on glycemic control [54].

According to the findings of our study, the levels of irisin in the serum were increased with Vit D supplementation [55]. This is supported by previous studies which showed that 12 months of Vit D supplementation led to a significant increase in serum irisin levels in albino Vit D-deficient rats and Vit D-deficient subjects, respectively [56]. However, a previous intervention involving healthy subjects found that a single dose of 100,000 IU of Vit D did not result in significant changes in serum irisin levels following 28 days of intervention [57]. The precise mechanism underlying the effect of Vit D on the secretion of irisin and expression of FNDC5 remains to be fully elucidated. However, a recent study has shown that Vit D supplementation and exercise can increase irisin levels in diabetic rats, indicating the potential therapeutic applications of Vit D supplementation, irisin administration, and exercise in the treatment of diabetes. This study also found that PTP1B expression was lower in the Vit D group. This is consistent with recent evidence suggesting that Vit D can act as a stimulator to decrease PTP1B activity and gene expression [58, 59]. The PTP1B gene is of particular significance in the context of insulin function and the treatment of T2D [60]. It has been identified as a regulator of insulin function in mammals and a pharmacological target for the treatment of T2D. The results of the current study demonstrate that Vit D in combination with exercise improves insulin resistance and reduces levels of blood glucose in T2D rats. These beneficial effects are associated with a decrease in the expression of PTP1B, an enzyme that plays a key role in insulin signaling by catalyzing the dephosphorylation of the insulin receptor (IR) [61]. The expression of PTP1B can be augmented by alterations in protein tyrosine kinases (PTKs) activity [62], which leads to impaired binding of insulin receptors to target tissues such as those observed in T2D, obesity, insulin resistance, and leptin deficiency [63]. Previous studies have also demonstrated that knocking out the PTP1B gene in rats is associated with reduced fatty tissue, increased energy consumption, and greater insulin sensitivity [64, 65]. Therefore, noninvasive interventions such as exercise or dietary modifications that lower PTP1B expression could help reduce insulin resistance levels, enhance insulin function, and improve glycemic control. To this end, the study found that the combination of AT and Vit D resulted in a greater reduction in PTP1B expression compared to AT or Vit D alone, potentially offering a promising intervention strategy.

The findings of the study suggest that decreasing the expression of PTP1B in adipose tissue through AT and Vit D intake can potentially result in lower insulin resistance and improved glycemic control. Prior studies have also shown that a reduction in PTP1B expression can affect insulin signaling pathways and improve insulin sensitivity. Specifically, PTP1B-deficient rats exhibit decreased TNF-α-dependent insulin resistance and increased insulin sensitivity in adipose tissue and skeletal muscles. Additionally, deleting PTP1B in the muscles of rats that consume a high-fat diet can improve glucose uptake and insulin signaling in skeletal muscles [66]. Previous research suggests that high levels of PTP1B induced by endoplasmic reticulum (ER) stress via ROS-NF-κB activation can increase proteins that stimulate insulin resistance in obesity. The AT and Vit D combination may reduce PTP1B levels and increase irisin levels in the serum, potentially leading to improved insulin function in target tissues probably by inhibiting ER stress induced by high-fat diets and free radicals. Therefore, decreased PTP1B expression may offer a promising intervention strategy for managing insulin resistance and glycemic control in T2D.

### Strengths and limitations of the study

Our research employed a randomized, placebo-controlled trial in an animal model of diabetes to investigate gene expression changes and had several strengths including no dropouts. However, it is too early to make conclusive statements. One of the limitations of our study was the absence of data on the expression of the FND5 gene in skeletal muscle and fat, which could have helped to determine the source of circulating irisin. Another limitation was the lack of evaluation of other significant transcriptional and posttranscriptional factors.

## Conclusion

Our study found that 8 weeks of AT with Vit D supplementation resulted in a reduction in HOMA-IR in rats with T2D. This decline was linked to decreases in body weight, BMI, and visceral fat, as well as an increase in serum irisin levels and improvement in PTP1B-dependent insulin function in adipose tissue in response to AT and Vit D. Nevertheless, more research is necessary to elucidate the genetic mechanisms underlying insulin function in response to AT with Vit D.

## Data Availability

The datasets generated and analyzed during the current study are not publicly available due to ongoing data analysis but are available from the corresponding author upon reasonable request.

## Abbreviations

AT: Aerobic training
BMI: Body mass index
ELISA: Enzyme-linked immunosorbent assay
ER: Endoplasmic reticulum
FI: Food intake
FNDC5: Fibronectin type 3 domain containing 5
FOXO1: Forkhead box O1
HOMA-IR: Homeostatic model assessment for insulin resistance
PGC-1α: Peroxisome proliferator-activated receptor-gamma coactivator-1 alpha
PPARγ: Peroxisome proliferator-activated receptor-gamma
PTKs: Protein tyrosine kinases
PTP1B: Protein tyrosine phosphatase 1B
T2D: Type 2 diabetes
TCF7L2: Transcription factor 7-like 2
Vit D: Vitamin D
